# Etiological Classification and Clinical Assessment of Children and Adolescents with Disorders of Sex Development

**DOI:** 10.4274/jcrpe.v3i2.16

**Published:** 2011-06-08

**Authors:** Sema Erdoğan, Cengiz Kara, Ahmet Uçaktürk, Murat Aydın

**Affiliations:** 1 Department of Pediatric Endocrinology, Ondokuz Mayis University, Samsun, Turkey

**Keywords:** Disorders of sex development, etiology, classification, children, adolescents

## Abstract

**Objective:** In 2006, the Lawson Wilkins Pediatric Endocrine Society (LWPES) and the European Society for Paediatric Endocrinology (ESPE) published a consensus statement on management of intersex disorders. The aim of our study was to determine the etiological distribution of disorders of sex development (DSD) according to the new DSD classification system and to evaluate the clinical features of DSDs in our patient cohort.

**Methods:** We retrospectively reviewed the records of patients followed up during the past three years. The subjects were divided into three etiologic groups according to their karyotypes. The definite diagnosesin each subgroup were established by clinical and laboratory investigations including abdominopelvic imaging as well as basal and stimulated hormone measurements. Molecular genetic testing, except for CYP21A2 gene, could not be performed.

**Results:** Out of a total of 95 patients, 26 had sex chromosome DSD, 45 had 46,XY DSD and 24 had 46,XX DSD. The most common causes of DSDs were Turner’s syndrome (TS), congenital adrenal hyperplasia (CAH) and androgen insensitivity syndrome (AIS). There was a wide variation in age of presentation ranging from 1 day to 17.5 years with a mean of 6.5±6.5 years. The most frequent complaints at presentation were ambiguous genitalia, isolated perineal hypospadias and short stature.

**Conclusion:** The results of our study demonstrate that the new DSD classification system leads to a major change in the distribution of etiological diagnoses of DSDs, which is exemplified by the significant frequencies of TS and vanishing testes syndrome. This alteration expands the clinical spectrum and increases the mean age at diagnosis. However, the most common causes of ambiguous genitalia, such as CAH and AIS, remain unchanged. Further studies using molecular genetic analyses are needed to give a more precise distribution of etiologies of DSDs, especially in 46,XY patients.

**Conflict of interest:**None declared.

## INTRODUCTION

Disorders of sex development (DSD) are defined as congenital conditions in which development of chromosomal, gonadal, or anatomical sex is atypical (1). In 2006, the Lawson Wilkins Pediatric Endocrine Society (LWPES) and the European Society for Paediatric Endocrinology (ESPE) published a consensus statement on management of intersex disorders and proposed the umbrella term ‘DSD’ instead of terms like ‘intersex’, pseudohermaphroditism (PH)’, ‘hermaphroditism’, ‘sex reversal’, which are often perceived as pejorative by patients and can be confusing to both health professionals and parents (2,3). Besides the new nomenclature, these two societies proposed a new classification system for causes of DSDs on the basis of karyotype analysis. The new DSD classification includes three main diagnostic categories: sex chromosome DSD, 46,XY DSD (formerly male PH) and 46,XX DSD (formerly female PH). The category of sex chromosome DSD embraces not only ovotesticular DSD (formerly true hermaphroditism) and 45,X/46,XY mixed gonadal dysgenesis, but also Turner’s syndrome (TS) and Klinefelter’s syndrome (KS), which are not included in the previous classifications of intersex disorders (4,5). Because the LWPES/ESPE consensus has taken the karyotype as the primary root, ovotesticular DSD has been classified in three DSD categories - XX, XY and XX/XY. The new classification also includes disorders such as vanishing testes syndrome and TS, which are not associated with genital ambiguity at birth. Thus, it would be expected that the inclusion of such entities into the new classification might lead to changes in the distribution of etiological diagnoses.

There are many studies on intersex disorders, whereas, to our knowledge, there are no data on the frequency of etiological diagnoses based on the new DSD classification system proposed by the LWPES/ESPE consensus group. Therefore, the aim of our study was to determine the etiological distribution of DSDs according to the new classification and to evaluate the clinical features of the most commonly encountered DSDs in our patient cohort.

## MATERIALS AND METHODS

We retrospectively reviewed the records of patients with DSD followed up during the past three years at the Department of Pediatric Endocrinology of Ondokuz Mayıs University. A detailed history including age at presentation, main complaints, sex of rearing, consanguinity, and family history of similar illness was taken for each patient. A thorough clinical examination consisting of anthropometry, assessment of pubertal stage, and presence of hyperpigmentation, hypertension, associated anomalies or dysmorphic features was made and recorded for each patient. Prader scoring system and external masculinization score (EMS) were used to determine the degree of external virilization in 46,XX and 46,XY DSD patients (6,7). Criteria suggesting DSD included overt genital ambiguity, apparent female genitalia with clitoromegaly, posterior labial fusion or inguinal/labial mass, and apparent male genitalia with non-palpable testes, micropenis, isolated perineal hypospadias or mild hypospadias with undescended testis (2). Also, file records of older children and adolescents who had incomplete or delayed puberty or primary amenorrhea were retrospectively evaluated with respect to DSD. Our study also comprised patients with diagnoses such as TS and KS, which are included in the new DSD classification. Patients with non-congenital (acquired) problems of late puberty were excluded from this study. 

As a part of routine evaluation of DSD, we performed karyotype analysis, abdominopelvic ultrasound (if required, magnetic resonance imaging), and hormone measurements including cortisol, 11-deoxycortisol (11-DOC), 17-hydroxyprogesterone (17-OHP), dehydroepiandrosterone sulfate (DHEA-S), androstenedione (A), testosterone (T), dihydrotestosterone (DHT), estradiol (E2) and gonadotropins. When it was necessary to demonstrate the presence or absence of functioning testicular tissue, as in the case of patients with suspected ovotesticular DSD and testicular dysgenesis or anorchia, the levels of anti-Müllerian hormone (AMH) and inhibin B were measured. We routinely analyze the common mutations in the CYP21A2 gene in congenital adrenal hyperplasia (CAH) patients. However, molecular genetic testing for mutations in androgen receptor (AR) gene and 5a-reductase gene in patients with defects in androgen action could not be performed. 

Vanishing testes syndrome was defined as normal virilized external genitalia, no testosterone response to hCG, and bilateral anorchia detected by imaging studies. Androgen insensitivity syndrome (AIS) was diagnosed in undervirilized males who had normal T and DHT response to hCG stimulation and absence of Müllerian structures. Those with normal female external genitalia were considered as complete AIS (CAIS) and the rest - as partial AIS (PAIS). Persistent Müllerian duct syndrome (PMDS) was defined as normal male external genitalia, normal response to hCG, and presence of Müllerian structures detected by ultrasound. In the 46,XX DSD group, the virilized females were first assessed with respect to basal (and when indicated, ACTH-stimulated) levels of adrenal steroids and androgens. Patients with elevated serum levels of 17-OHP and T were diagnosed as CAH. The diagnosis of CAH due to 21-hydroxylase deficiency was established by a serum 17-OHP level greater than 100 ng/mL (300 nmol/L) after the first 48 hours and was confirmed by CYP21A2 gene analysis. Patients with markedly elevated serum levels of 11-DOC and DHEA-S together with moderately increased serum 17-OHP levels were accepted as 11b-hydroxylase and 3b-hydroxysteroid dehydrogenase deficiencies, respectively. Virilized females with normal 17-OHP and T levels were categorized as maternal androgen excess (such as luteoma) or congenital structural abnormality. In these cases, to exclude ovotesticular DSD, serum levels of AMH and inhibin B were measured, and imaging of the internal genitalia and gonads was performed. Gonadal (ovarian) dysgenesis was diagnosed in 46,XX DSD patients who presented with absent breast development or primary amenorrhea, high gonadotropin levels, and streak (underdeveloped) gonads detected by imaging studies.

The data were analyzed using SPSS software (Statistical Package for the Social Sciences, version 15; SPSS Inc., Chicago, IL, USA). The results are given as mean±SD or median (range) values, and as percentages, where appropriate. Comparison between PAIS patients reared as male and those raised as female regarding EMS was made using the Mann-Whitney U test. A p value of less than 0.05 was considered statistically significant.

## RESULTS

**Figure 1:**
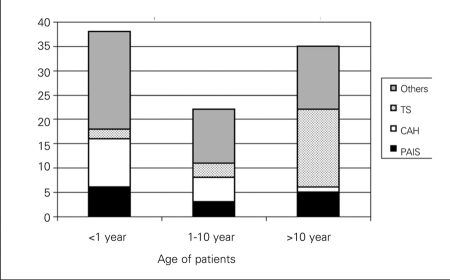
The age distribution of DSD patients according to their etiologic diagnoses The most frequent diagnoses of the 95 patients with disorders of sex development (DSD) were Turner’s syndrome (TS, n=21) congenital adrenal hyperplasia (CAH, n=16) and partial androgen insensitivity syndrome (PAIS, n=14). While TS patients were mostly diagnosed in adolescence, patients with salt-wasting and simple virilizing CAH were usually seen at infancy and childhood, respectively. PAIS and other DSD patients were observed in all age groups, with a predominance in infancy

A total of 95 patients met the criteria for DSD. There was a wide variation in age at presentation ranging from 1 day to 17.5 years with a mean age of 6.5±6.5 years. Thirty eight patients presented in infancy, 22 - between 1 and 10 years, and 35 - at ages older than 10 years (Figure 1). A history of consanguinity was obtained in 17 cases (18%). The consanguinity rate in CAH patients (43%) was higher than that in the entire study group. At the time of presentation, while 37 patients - newborns and young infants - were not yet assigned a sex of rearing, in the older age groups, 22 patients were raised as males and 36 as females. A total of 9 patients with a 46,XY karyotype (5 - PAIS, 3 – CAIS, and 1 - 5a-reductase eficiency) had been reared as females, and 2 CAH patients with 46,XX karyotype as males. All patients in the series were assigned female or male sex by a gender assessment team composed of pediatric specialists in endocrinology, surgery, urology and psychiatry as well as medical geneticist. After gender (re)assignments, the total study group contained 42 males and 53 females. The main complaints at presentation in DSD patients were ambiguous genitalia (n=23), short stature (n=19), isolated perineal hypospadias (n=9), primary amenorrhea (n=8), late or incomplete puberty (n=8), micropenis (n=6) and clitoromegaly (n=5). Out of a total of 95 patients, 26 had sex chromosome DSD, 45 had 46,XY DSD and 24 had 46,XX DSD (Table 1). TS (n=21) was the most common cause (80%) of sex chromosome DSD. There were a few patients with sex chromosome DSD other than TS, i.e. 45,X/46,XY mixed gonadal dysgenesis (n=3), 45,XX/46,XY ovotesticular DSD (n=1) and 47,XXY, KS (n=1). Patients with mixed gonadal dysgenesis had overt ambiguous genitalia together with a normal testis on one side and a streak (dysgenetic) gonad on the contralateral side. These patients were assigned male sex and their dysgenetic gonads were removed. Histological examination did not reveal any sign of germ cell malignancies. A patient presenting with genital ambiguity had Müllerian structures and bilateral ovotesticular gonads on histological examination. Bilateral gonadectomy was performed in a 46,XX/46,XY DSD patient who had been reared and assigned as female. Gonadoblastoma or germinoma were not detected in the removed gonads. The patient with KS presented with delayed puberty.

Fifty three percent of patients with TS had 45,X karyotype, and the remaining had various karyotype abnormalities such as mosaicism (45,X/46,XX;14%), isochromosome (45,X,i(Xq); 10%) and ring X chromosome (46,X,r(X); 5%). The mean age at diagnosis for TS patients was 12.1±4.9 years (from intrauterine period to 17.5 years). Of 21 patients with TS, 16 (76%) had presented at ages older than 10 years with main complaints of short stature and primary amenorrhea (Figure 1). Three patients, of ages between 1 and 10 years, presented with short stature. One patient was diagnosed in the newborn period with lymphoedema of the feet, and another one - incidentally at amniocentesis. The mean height SD score of the TS patients was -3.82±0.75. Pelvic ultrasound revealed small or no ovarian tissue in all TS patients. Plasma levels of follicle-stimulating hormone, luteinizing hormone and E2 were 97.3±41.9 (24-170) mIU/mL, 19.7±10.7 (0.15-44) mIU/mL and 11.9±4.4 (5.3-21) pg/mL, respectively. 

Defects in androgen action [AIS (14 partial, 3 complete) and 5a-reductase deficiency (n=3)] constituted the largest fraction (44%) of the 46,XY DSD group. Isolated perineal hypospadias (n=9), vanishing testes syndrome (n=6), micropenis with mild hypospadias or cryptorchidism (n=6) and PMDS (n=2) were among other diseases in this group (Table 1). Two patients’ genital ambiguity was associated with unidentified syndromes. In our study group, there was no case with 46,XY gonadal dysgenesis or T biosynthesis defect.

The mean age at diagnosis for PAIS patients was 5.7±5.9 years (1 day-15.4 years). The ages at presentation were under one year in 6 patients, between 1 and 10 years in 3 patients, and above 10 years in 5 patients (Figure 1). In both infancy and childhood, the main complaint at presentation was overt genital ambiguity. However, adolescent patients with PAIS presented with clitoromegaly, amenorrhea, and incomplete puberty and had already been reared as females. In addition, three CAIS patients raised as females had presented with incomplete puberty or primary amenorrhea after 10 years of age. Signs related to some androgenic effects, such as pubic/axillary hair and phallic enlargement, differentiated the females with PAIS from those with CAIS. Breast development and amenorrhea were seen as common clinical features in both patient groups. Median value of EMSs was 7 (range: 2-10; normal: 0-12) in all PAIS patients. The median EMS (8.5; range: 4-10) for PAIS patients reared as male was significantly higher than that (3; range: 2-5) for PAIS patients raised as female (p<0.001). These scores in patients with CAIS ranged from 1 to 2, representing the presence of abdominal or inguinal testicles with normal female external genitalia (7). In the entire AIS group, plasma levels of basal and hCG-stimulated T were 1.17±1.60 (0.08-5.90) ng/mL and 4.88±2.91 (0.54-12.5) ng/mL, respectively. In all patients with AIS except two, serum peak T levels were higher than 1 ng/mL. In two patients whose hCG-stimulated T levels were 0.54 and 0.85 ng/mL, the T/A ratios were 2.6 and 3.2, respectively. These values were not considered as suggestive of 17b-HSD deficiency. Also, these patients were not considered as gonadal dysgenesis because of normal age-matched serum AMH and inhibin B levels and normal-appearing testes by imaging studies. On the other hand, these two patients were older children who had undescended testes; thus, incomplete responses to hCG might have been a result of Leydig cell dysfunction secondary to cryptorchidism. After hCG stimulation, the mean T/DHT ratio was 10.1±8.2 (2.5-29.1) in the PAIS/CAIS groups. In three patients accepted as 5a-reductase deficiency, these ratios were 36.3, 54.6 and 66.5. In addition, serum levels of hCG-stimulated T and T/DHT ratios were normal in the other 46,XY DSD patients who had isolated perineal hypospadias (n=9) and micropenis associated with mild glandular hypospadias or cryptorchidism (n=6). In 46,XY DSD group, there was no patient with congenital hypogonadotropic hypogonadism.

The majority of patients with 46,XX DSD had CAH, consisting of 21-hydroxylase (n=14), 11b-hydroxylase (n=1) and 3b-hydroxysteroid dehydrogenase (n=1) deficiencies. Four patients presenting with delayed puberty or primary amenorrhea were diagnosed as 46,XX gonadal dysgenesis. In the other four mildly virilized newborn females who had isolated clitoromegaly (n=2) or clitoromegaly with partial labial fusion (n=2), serum hormone measurements (including 17-OHP, DHEA-S, T, androstenodione, AMH and inhibin B) were in normal ranges, and abdominopelvic imaging also revealed normal internal genitalia and gonads. In this group, there was no history of maternal drug intake during pregnancy; however, one mother had experienced temporary excessive hair growth and developed acne during the first trimester. The reason of androgenic effect in these patients could not been completely understood. In a patient whose mother had been mildly androgenized during pregnancy, the probable cause of virilization was considered to be a luteoma. The other three patients were categorized as congenital structural abnormality and were followed up (Table 1). 

Out of 14 females with CAH due to 21-hydroxylase deficiency, 9 had salt-wasting type and 5 had simple virilizing type. The median age at diagnosis for salt wasters was 5 (1-25) days. Patients with simple virilizing CAH presented at median age of 5.3 (1.6-13.1) years with previously unrecognized genital ambiguity and/or virilization. Two patients from the latter group had been reared as male sex. The oldest one had heterosexual pseudopuberty and short stature. Hyperpigmentation was detected in six patients. Median value of Prader scores for patients with 21-hydroxylase deficiency was 4 (range: 3-5). Plasma levels of 17-OHP and T at diagnosis were 427±332 (227-1200) ng/mL and 6.4±5.9 (0.8-16) ng/mL, respectively. A patient with 11b-OH deficiency had presented at 5.5 years of age with unrecognized genital ambiguity (Prader 3 virilization), pubic hair and hypertension. Serum levels of 17-OHP, T and 11-DOC were 54 ng/mL, 1.1 ng/mL and >25 ng/mL, respectively. A female patient presenting with salt wasting and mild clitoromegaly (Prader 2 virilization) at 3 months of age was diagnosed with CAH due to 3b-hydroxysteroid dehydrogenase deficiency because of increased serum levels of DHEA-S (673 μg/dL), 17-OHP (25 ng/mL) and T (0.87 ng/mL).

**Table 1:**
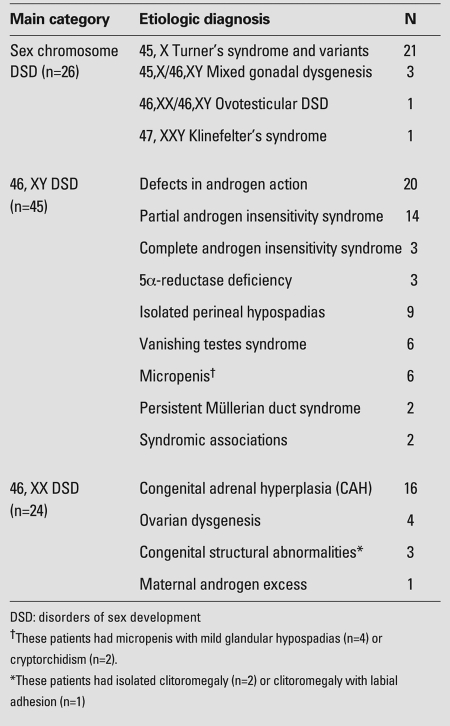
Etiological classification in 95 patients with DSD

## DISCUSSION

In 2006, the LWPES and the ESPE published a consensus statement on the management of ‘intersex’ disorders and proposed a new classification for DSD (2,3). To our knowledge, to date, there is no study regarding the etiological classification of DSDs according to the new consensus document. Therefore, this is the first study evaluating the etiological diagnoses of DSDs according to the new classification, and we believe it provides some interesting data.

Firstly, our data demonstrate that the most common causes of DSDs were TS, CAH and PAIS and, that patients with DSD may present at a wide age range varying from the first day of life to late adolescence. Our results also indicate that the clinical manifestations of DSDs are not limited to ambiguous genitalia, but show a broad spectrum including isolated hypospadias, micropenis, clitoromegaly, incomplete puberty, amenorrhea and, even, short stature. It is well known that PAIS and CAH are ‘intersex’ disorders associated with genital ambiguity, whereas TS and 46,XX gonadal dysgenesis are not. The results of our study demonstrate that the inclusion of TS and ovarian dysgenesis into the new etiologic classification of DSD has been the main factor that expands the clinical spectrum and increases the average age at diagnosis.The generic term DSD was proposed and defined as congenital conditions in which development of chromosomal, gonadal or anatomical sex is atypical (1,2,3). This nomenclature, which is recommended instead of the word ‘intersex’ referring primarily to xternal genital ambiguity, may be confusing in the clinical evaluation of patients. TS, the most frequent diagnosis in our DSD cohort, is a condition in which both chromosomal and gonadal sex are abnormal despite normal female external genitalia. Girls with TS are usually diagnosed in late childhood or adolescence when they are investigated for short stature or delayed puberty. Evaluation of patients with only ambiguous genitalia for DSD may cause some DSD patients to be overlooked. Therefore, our study indicates that a number of subjects with short stature and late puberty, who especially have some dysmorphic features consistent with TS, possibly will be identified as DSD patients.

TS is one of the most common sex chromosome abnormalities with an incidence of 1:2000 to 1: 5000 in live-born females (10). Thus, it appears reasonable that in this cohort, TS also constitutes a significant proportion of DSD cases. In addition, it is noteworthy that we have found only one patient with KS in our DSD cohort. The number of patients with KS as compared to those with TS is very low, given its reported incidence ranging between 1:500 and 1:1000 live births (11). However, while girls with TS usually present with short stature in the prepubertal period or with primary amenorrhea at puberty, the diagnosis of KS is rarely made before puberty because of paucity or subtleness of clinical manifestations in childhood. It is likely that a significant proportion of children with mosaic forms of KS or with milder phenotypes may not have been diagnosed in the age group covered in this study. 

Approximately fifty percent of the patients in the cohort had 46,XY DSD. This finding is consistent with other studies (12,13,14). The most common cause of 46,XY DSD was AIS, either PAIS or CAIS, representing 44% of 46,XY patients. The percentage of PAIS patients in our DSD cohort is moderately higher than the results of previous studies (12,13,14), which can be partly attributed to the fact that we have used a T/DHT ratio as high as 30 to differentiate 5a-reductase deficiency from AIS. A few patients who had a value less than 30 might have had 5a-reductase deficiency. In addition, our 46,XY DSD group included patients with isolated perineal hypospadias and micropenis without overt genital ambiguity. In these patients, we ruled out a probability of Leydig cell dysfunction. Such subjects may have been either cases of isolated anatomical defect of unknown etiology or may represent the milder end of the spectrum of PAIS (9). Therefore, in the 46,XY DSD patients who have normal T production, diagnoses of AIS and 5a-reductase deficiency need to be confirmed by molecular genetic analyses. 

On the other hand, the identification of a causative AR mutation in PAIS population is rare (12). In fact, it is possible that patients with CAIS or PAIS in whom no mutations were found in the AR gene may carry a mutant coactivator protein to explain the androgen resistance (9). Even though one of the major limitations encountered in our study is the inability to make definite etiological diagnoses for 46,XY DSD patients, it is true that undermasculinized patients with normal androgen production (demonstrated by hCG stimulation) have been exposed to insufficient androgen effects, regardless of the cause (failure to produce DHT or failure to respond to androgens). Therefore, the findings of this study suggest that 46,XY DSD mostly results from defects in androgen action and that androgen biosynthesis defects are rarely seen. However, we have to emphasize that we have not done genetic analysis in the presumed AIS cases. 

Interestingly, we observed that the frequency of vanishing testes syndrome among 46,XY DSD patients was notably high. These patients presenting with undescended testes had no genital ambiguity. Because vanishing testes syndrome was not in the former etiological classifications of intersex disorders (4,5), its frequency within DSD is unknown. Cryptorchidism, together with hypospadias, is among the most common genitourinary anomalies in male children, affecting 1.6% to 9% live births (15). In a recent study, the prevalence of cryptorchidism at birth was found to be 5.9%, and approximately one fourth of the patients had non-palpable testes (16). Another new population-based study carried out in boys aged less than one year to 19 years has established that the prevalence of cryptorchidism was 1.52% (17). A very recent study has shown that about half of the patients with impalpable testes had vanishing testes syndrome (18). Therefore, we consider that the finding of remarkable frequency of vanishing testes syndrome in the new DSD classification system is not unexpected. In the 46,XX DSD group, the most common condition was CAH due to 21-hydroxylase deficiency, a finding compatible with its worldwide incidence of 1:14 000 live births (19). While CAH was identified to occupy second place in terms of frequency following TS, it was the most common etiology in patients presenting with genital ambiguity. In fact, CAH is the commonest cause of ambiguous genitalia of the newborn (20). In an epidemiological study, the incidence of ambiguous genitalia in neonates was identified as 1:5 000 births, and the most common diagnosis was CAH, followed by AIS and mixed gonadal dysgenesis (21). Our results are in agreement with this study and suggest that CAH remains the most common cause of ambiguous genitalia, regardless of the classification system used in DSD.In conclusion, this study reveals that the new DSD classification system leads to a major change in the distribution of etiological diagnoses of DSDs, which is exemplified by the significant frequencies of TS and vanishing testes syndrome. However, the most common causes of ambiguous genitalia such as CAH and AIS remain unchanged. Further studies using molecular genetic analyses are needed to give a more precise distribution of etiologies of DSDs, especially in 46,XY patients.
